# Apaf1 plays a negative regulatory role in T cell responses by suppressing activation of antigen-stimulated T cells

**DOI:** 10.1371/journal.pone.0195119

**Published:** 2018-03-29

**Authors:** Honglian Tong, Yasunobu Miyake, Fumika Mi-ichi, Yoichiro Iwakura, Hiromitsu Hara, Hiroki Yoshida

**Affiliations:** 1 Division of Molecular and Cellular Immunoscience, Department of Biomolecular Sciences, Faculty of Medicine, Saga University, Saga, Japan; 2 Center for Experimental Animal Models, Institute for Biomedical Sciences, Tokyo University of Science, Chiba, Japan; The University of Texas MD Anderson Cancer Center, UNITED STATES

## Abstract

Apaf1 is a critical component of the apoptosome and initiates apoptosis downstream mitochondrial damages. Although the importance of Apaf1 in embryonic development was shown, the role of Apaf1 in immune responses, especially T cell responses, has yet to be elucidated. We generated T cell-specific Apaf1-deficient mice (Lck-*Cre*-*Apaf1*^f/f^ mice) and examined the antigen-specific delayed-type hypersensitivity (DTH). Lck-*Cre*-*Apaf1*^f/f^ mice exhibited exacerbation of DTH responses as compared with Apaf1-sufficient control mice. In Lck-*Cre*-*Apaf1*^f/f^ mice, antigen-specific T cells proliferated more, and produced more inflammatory cytokines than control T cells. Apaf1-deficient T cells from antigen-immunized mice showed higher percentages of activation phenotypes upon restimulation *in vitro*. Apaf1-deficient T cells from naive (non-immunized) mice also showed higher proliferation activity and cytokine production over control cells. The impact of Apaf1-deficiency in T cells, however, was not restored by a pan-caspase inhibitor, suggesting that the role of Apaf1 in T cell responses was caspase-independent/non-apoptotic. These data collectively demonstrated that Apaf1 is a negative regulator of T cell responses and implicated Apaf1 as a potential target for immunosuppressive drug discovery.

## Introduction

Immune responses include activation and clonal expansion of relevant lymphocyte populations to wipe up invading foreign antigens. Once the antigens are removed, circulating activated lymphocytes, especially T cells, should be eliminated to avoid unnecessary inflammation and to maintain immune homeostasis, while only a few of the activated lymphocytes are fated to develop into memory lymphocytes. In this process, apoptosis plays critical roles[1]. Apoptosis in T cells can be initiated either extrinsically or intrinsically[2]. The extrinsic pathway is initiated by ligation of cell surface death receptors, including Fas[3]. The intrinsic pathway involves mitochondria, where activation of pro-apoptotic members of Bcl-2 family induces the release of cytochrome *c* (Cyt *c*) resulting in the formation of apoptosome composed with Cyt *c*, caspase (Casp) 9, and Apaf1[4]. In both pathways, activation of effector caspases, including Casp3, 6, and 7, downstream either death receptors or apoptosome, leads to degradation of cellular proteins and resultant cell death. While caspase-dependent apoptosis occurs in activated T cells or in T cells deprived of survival factors, caspase activation is reportedly dispensable for thymocyte development and for T cell homeostasis, implying caspase-independent cell death also occurs in T cells[5, 6].

Apaf1, the mammalian homolog of *C*. *elegans* CED-4, participates in the formation and activation of the apoptosome[7, 8]. Absence of Apaf1[9, 10], Casp9[11, 12] or Apaf1-activating form of Cyt *c*[13], leads to the failure of mitochondria-dependent apoptosis. The result is remarkable accumulation of neurons during embryonic brain development, demonstrating the critical role of Apaf1 and other components of the apoptosome in development[14]. Roles of Apaf1 in thymocytes/T cell apoptosis have been shown in a couple of previous reports; while Apaf1-deficient thymocytes were resistant to apoptosis induced by mitochondria-insulting stimulations, such as γ-irradiation and corticosteroid treatment, they were sensitive to Fas-induced apoptosis just like Apaf1-sufficient thymocytes[10]. In addition, successful negative selection of Apaf1-deficient thymocytes showed that Apaf1-dependent apoptosis was not required for the negative selection of thymocytes[6]. Although most of the Apaf1-deficient mice showed perinatal lethality due to the abnormal development of the brain, there were a few survivors, which showed no abnormal accumulation of peripheral lymphocytes, indicating that Apaf1 was not required for the elimination of auto-reactive T cells in the periphery or in the homeostasis of peripheral T cells in number (H. Y., unpublished data). The roles and functions of Apaf1 in the regulation of T cell-mediated immune responses, however, have yet to be clarified.

In this study, we took advantage of T cell-specific Apaf1-deficient mice, Lck-*Cre*-*Apaf1*^f/f^, in which *Apaf1* gene was disrupted with *Lck* promoter-driven *Cre* expression, to investigate the biological function of Apaf1 in T cells. Apaf1-deficient T cells showed resistance to mitochondria-dependent apoptosis but showed susceptibility to Fas-mediated apoptosis. We then performed the delayed-type hypersensitivity (DTH) assay, using ovalbumin (OVA)-specific T cell receptors (TCR)-expressing mice (OTII mice), and found that antigen-specific T cell activation leads to enhanced proliferation and Th1-type immune responses in Lck-*Cre*-*Apaf1*^f/f^-OTII mice, as compared with control Apaf1-sufficient OTII mice. Apaf1-deficient T cells showed higher percentages of cells expressing early activation markers after stimulation than control T cells. Surprisingly, Apaf1-sufficient T cells treated with a pan-caspase inhibitor, z-VAD-*fmk* (carbobenzoxy-valyl-alanyl-aspartyl-[O-methyl]- fluoromethylketone), did not reproduce the activation-related phenotypes observed in Apaf1-deficient T cells, indicating caspase-independent roles of Apaf1 during T cell activation. Our data suggested that Apaf1 in T cells is a negative regulator of immune responses.

## Materials and methods

### Generation of T cell-specific Apaf1-deficient mice

The design of the conditional targeting vector for *Apaf1* is shown in S1 Fig, in which exons 2 and 3 are flanked by two *loxP* sites. The linearized targeting vector was electroporated into E14K ES cells and homologous recombinants were selected. The heterozygous *Apaf1* mutant (*Apaf1*^f-Neo/+^) ES clones were injected into C57BL/6 blastocysts to generate chimeric mice. *Apaf1*^f-Neo/+^ mice were selected and crossed into C57BL/6 mice more than 10 times; the PGK-neo cassette was removed by mating the heterozygotes with CAG-*Flpe* transgenic (Tg) mice (RBRC01834, RIKEN BRC). Mice heterozygous for *Apaf1* mutation (*Apaf1*^f/+^) were then crossed with Lck-*Cre* Tg mice and transgene-positive *Apaf1*^f/+^ mice were intercrossed to generate Lck-*Cre*-*Apaf1*^f/f^ mice and Lck-*Cre*-*Apaf1*^f/+^ mice. Lck-*Cre*-*Apaf1*^f/f^ -OTII mice were similarly generated by crossing Lck-*Cre*-*Apaf1*^f/f^ mice with OTII-Tg mice expressing OVA-specific TCR. Lck-*Cre* Tg mice and OTII mice were kindly provided by Dr. A. Yoshimura, Keio University, Japan. Successful disruption of *Apaf1* gene was confirmed with genomic Southern blot analysis and absence of Apaf1 protein in Lck-*Cre*-*Apaf1*^f/f^ T cells was confirmed with Western blot analysis using anti-Apaf1 antibody (PharMingen)

### Animal care

This study was carried out in strict accordance with the recommendations in the Guide for the Care and Use of Laboratory Animals of the National Institutes of Health. The protocol was approved by the Institutional Animal Care and Use Committee of Saga University (Approval number 27-047-0). All efforts were made to minimize suffering.

### Apoptosis assay

For induction of apoptosis in thymocytes, thymocytes (5 × 10^5^ cells/well) were cultured in 96-well plates with or without the following agents or stimulation; dexamethasone (10, 30, 100 nM. Sigma), staurosporine (100, 300, 1000 nM. Sigma), γ-irradiation (100, 300, 1000 cGy), anti-Fas antibody (clone Jo2; 1 μg/ml. PharMingen) plus cycloheximide (0.2, 1 μg/ml. Sigma). After 20 hours, apoptotic cells were detected by staining with Annexin V- FITC (PharMingen) and propidium iodide (PI, BioLegend).

For activation-induced apoptosis of peripheral lymph node (LN) T cells, LN cells were stained with FITC-labeled anti-Thy1.2 antibody (eBioscience) followed by positive selection procedure using anti-FITC magnetic beads (MACS; Miltenyi Biotec) and LS columns (Miltenyi Biotec). Purified T cells (>90% T cells, 3× 10^6^ cells/well) were activated with plate-bound anti-CD3ε (0.3 μg/ml; clone 145-2C11, eBioscience) plus anti-CD28 antibodies (3 μg/ml; clone 37.51, eBioscience) for 48 hours in 6-well plates. After removal of apoptotic cells by staining the cells with Annexin V-APC followed by anti-APC magnetic beads (MACS) procedure, remaining cells (Annexin V-negative cells >97.6%) were cultured (2 × 10^5^ cells/200 μl/well) either in fresh medium alone, or conditioned medium prepared from the supernatants of the primary stimulation culture (anti-CD3ε plus anti-CD28 antibodies), or with anti-CD3ε antibody in fresh medium. Twenty-four hours later, apoptotic cells were detected as above.

### Cell activation and cytokine production

Mice were subcutaneously (s.c.) immunized with 200 μg of OVA emulsified in complete Freund’s adjuvant (CFA, Difco) at the tail base. Inguinal LNs were collected and single cell suspensions were prepared. Total LN cells (2 × 10^5^ cells/well) were cultured in the presence of OVA protein (0, 1, 10, 100 μg/ml) or anti-CD3ε antibody (1 μg/ml) in RPMI1640 medium supplemented with 10% fetal bovine serum. For proliferation assay, cells were pulsed with 1 μCi/well of ^3^H-thymidine (PerkinElmer) during the last 7 hours of 48 hours of culture and incorporated radioactivity was measured. Culture supernatants were removed before ^3^H-thymidine pulse and IL-2, IFN-γ or IL-17 levels in the supernatants were measured using ELISA Ready-SET-Go (eBioscience). In some experiments, a pan-caspase inhibitor z-VAD-*fmk* (10 and 100 μM, MBL) was added into the culture.

### DTH assay

Seven days after immunization with OVA as above, mice were challenged s.c. at right footpad with 200 μg of OVA in 20 μl PBS. As a control, the same volume of PBS was injected into left footpad. Footpad thickness was measured with a dial vernier caliper (Teclock) on day 1 and 2. The magnitude of the DTH response was calculated as follows; footpad swelling (μm) = thickness of OVA-injected footpad − thickness of PBS-injected footpad. For histological analysis of the DTH lesions, paws were removed on day 2 and fixed with 10%-formaldehyde neutral buffer solution (Nacalai). After decalcification by a standard protocol, specimens were embedded in paraffin and were stained with hematoxylin-eosin (H&E). For analysis of the tissue-infiltrating cells, paws were thoroughly minced with scissors and then were incubated at 37°C for 1 hour in Hank's solution containing 1.0 mg/ml collagenase II (Worthington), 1.0 mg/ml dispase (Sigma-Aldrich) and 40 μg/ml Dnase I (Roche). After removing debris with 70 μm cell-strainers, cells were re-suspended into 33.7% Percoll (GE Healthcare) and pelleted by centrifugation at 1,000 *g*, for 20 minutes at 24°C. Cells were stained with anti-CD45.2-APC (eBioscience), anti-CD11b-FITC (eBioscience), anti-Ly6G-PE (BD PharMingen), anti-CD3ε-APC (eBioscience) antibodies, and analyzed by flow cytometry for expression of respective molecules.

### Carboxy-fluorescein-diacetate succinimidylester (CFSE) staining

Naïve LN cells were suspended in PBS at 2 × 10^7^ cells/ml and incubated with CFSE (DOJINDO) at a final concentration of 5μM CFSE plus 0.1%BSA for 10 min at 37°C, followed by washing 3 times with RPMI1640 medium. Stained cells (2 × 10^5^ cells/well) were cultured with anti-CD3ε or OVA Peptide (323–339, Peptides International) for 4 days, and evaluated for CFSE intensity.

### Flow cytometry

For detection of apoptotic cells, cells were stained as above. For detection of CD69, CD44, and CD62L, LN cells isolated from naïve mice or mice on day 8 after immunization were stimulated with OVA protein (0, 1, 10, 100 μg/ml) or anti-CD3ε antibody (1 μg/ml) and stained with anti-CD69 (PharMingen), anti-CD44 (TONBO Biosciences), and anti-CD62L (BD Biosciences) antibodies on day 0 or 2. For detection of OTII-TCR-Tg-positive cells, cells were stained with anti-TCR Vα2-FITC antibody (clone B20.1, BioLegend). Stained cells were analyzed with BD FACSVerse^TM^ (BD Biosciences) for detection of respective surface molecules.

### Western blot analysis

For detection of capase cleavage during T cell activation, inguinal LN cells were isolated from mice on day 8 after immunization and were stimulated with OVA protein (0, 10, 100 μg/ml) or anti-CD3ε antibody (1 μg/ml) as above with or without pan-caspase inhibitor z-VAD-*fmk* (10 and 100 μM). Cell lysates were prepared, electrophoresed, and blotted. Tubulin, caspases 3, 7, and 9 were detected with respective antibodies (anti-tubulin; Sigma Aldrich and anti-caspases; Cell Signaling Technology) and visualized using an enhanced chemiluminescence procedure (ImmunoStar LD, Wako).

### Statistical analysis

Experiments were repeated at least three times. Values were expressed as means + SD. Differences between control (Apaf1-sufficient) and Apaf1-deficient samples were analyzed using unpaired *t*-tests. Differences between two samples among multiple samples with various experimental conditions were analyzed using unpaired *t*-tests with Bonferroni correction (See figure legends). Alpha value was set at 0.05 and p values below the alpha value (0.05) were considered to be statistically significant.

## Results

### Generation of T cell specific Apaf1-deficient mice

**Fig 1:**
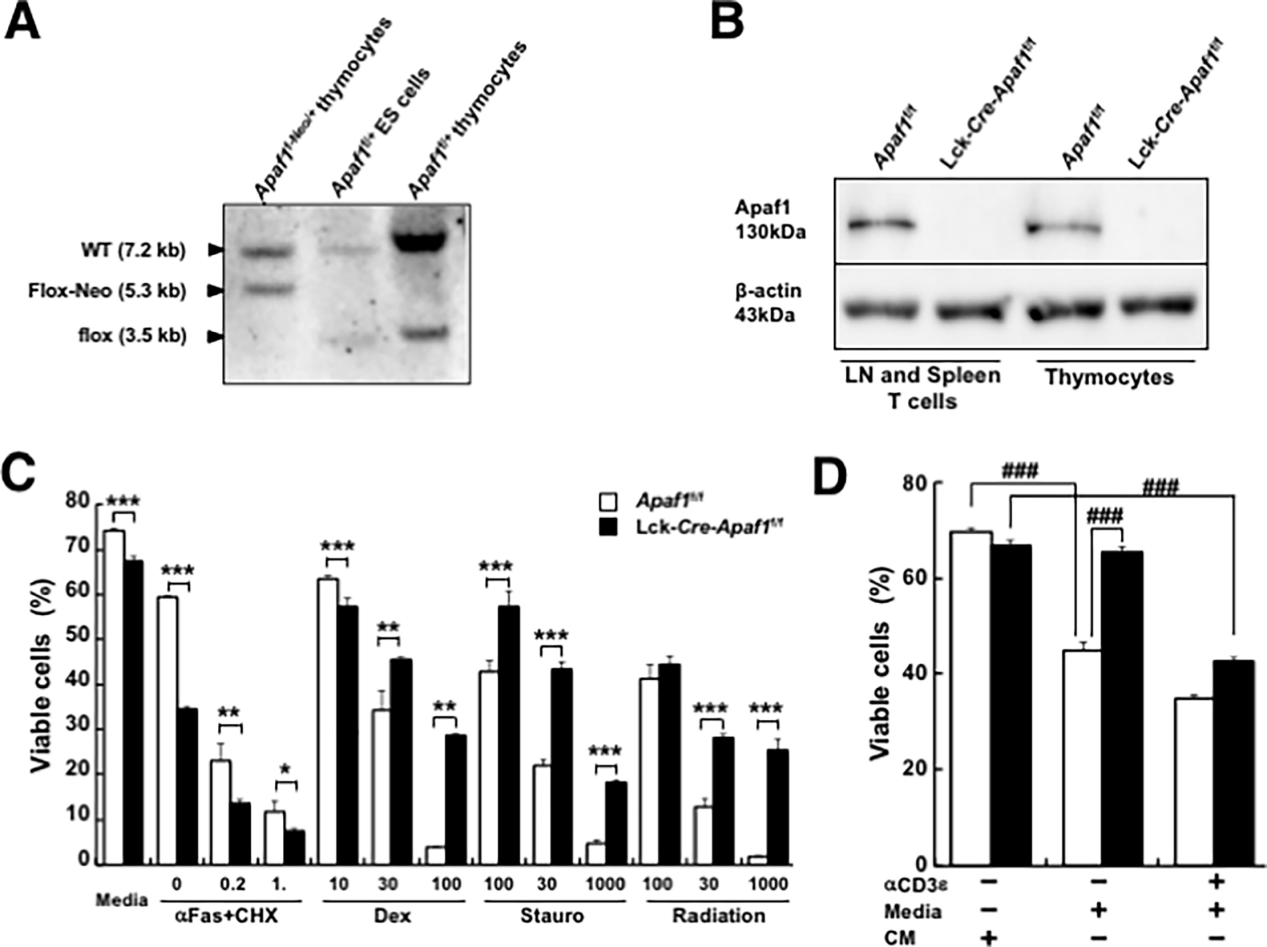
Generation of T cell-specific Apaf1-deficient mice. (A) Southern blot analysis of genomic DNA from *Apaf1*^f-Neo/+^ thymocytes (left), *Apaf1*^f/+^ thymocytes (right), and *Apaf1*^f/+^ ES cells, in which Neo gene was removed by transient expression of FLPe recombinase (middle). DNA was digested with EcoRI and detected with the probe at exon 4. (See S1 Fig for detail.) (B) Western blot analysis of proteins from LN T cells and thymocytes of *Apaf1*^f/f^ mice and Lck-*Cre*-*Apaf1*^f/f^ mice. (C) Thymocytes from *Apaf1*^f/f^ mice (open columns) or Lck-*Cre*-*Apaf1*^f/f^ mice (closed columns) were stimulated with indicated doses of anti-Fas antibodies plus cycloheximide (αFas + CHX), dexamethasone (Dex), staurosporine (Stauro), γ-irradiation, or left untreated. Apoptotic cells were detected by flow cytometry. (D) Purified T cells from LN of *Apaf1*^f/f^ (open columns) or Lck-*Cre*-*Apaf1*^f/f^ (closed columns) were activated for 48 hours with anti-CD3ε antibody plus anti-CD28 antibody. Activated cells, after removal of dead cells, were cultured in the presence of conditioned medium (CM), in the fresh medium for growth factor deprivation (Media), or re-stimulated with anti-CD3ε antibody in fresh medium for activation-induced cell death, for 20 hours. Apoptotic cells were detected by flow cytometry. Data show means + SD of triplicated samples. Experiments were repeated three times with similar results. *; p<0.05, **; p<0.01, and ***; p<0.002 compared between *Apaf1*^f/f^ and Lck-*Cre*-*Apaf1*^f/f^ cells, *t*-tests. ###; p’ (corrected p value) < 0.002, *t*-tests with Bonferroni correction.

T cell-specific Apaf1-deficient mice (Lck-*Cre*-*Apaf1*^f/f^ mice) were generated as described in the Materials and methods. Genotypes of the mice were confirmed by Southern blot analysis of the genomic DNA using two external probes (not shown) and a probe at exon 4 (Fig 1A) and *Cre*-driven successful ablation of Apaf1 in T cells and thymocytes were confirmed by Western blot analysis (Fig 1B). Mice sufficient or deficient for Apaf1 in T cells were viable, healthy, and fertile and showed no sign of brain deformity (data not shown). Lck-*Cre*-*Apaf1*^f/f^ mice showed no sign of anatomical and pathological abnormality including lymphocyte accumulation in the lymphoid organs (not shown). In addition, total numbers of thymocytes and cellularity of CD4^+^ and CD8^+^ thymocytes were comparable between Lck-*Cre*-*Apaf1*^f/f^ and control *Apaf1*^f/f^ mice (not shown). In our previous report, Apaf1-deficient thymocytes showed resistant to drug-induced and irradiation-induced apoptosis but showed susceptibility to Fas-induced apoptosis over WT thymocytes[10]. As expected, thymocytes from Lck-*Cre*-*Apaf1*^f/f^ mice showed similar resistance to dexamethasone-, staurosporine-, and irradiation-induced apoptosis over control thymocytes from *Apaf1*^f/f^ mice but showed susceptibility to Fas-induced apoptosis just like Apaf1-sufficient thymocytes (Fig 1C). Activated Lck-*Cre*-*Apaf1*^f/f^ peripheral T cells were refractory to the mitochondria-dependent passive apoptosis induced by growth factor deprivation as compared with Apaf1-sufficient *Apaf1*^f/f^ T cells (Fig 1D and S2 Fig); when LN T cells that had been previously activated with anti-CD3ε antibody plus anti-CD28 antibody were placed in new medium, in which T cell-derived IL-2 was insufficient, control *Apaf1*^f/f^ T cells showed significant reduction in viability whereas Lck-*Cre*-*Apaf1*^f/f^ T cells showed higher viability. When activated T cells were re-stimulated with anti-CD3ε antibody in a new medium for activation-induced cell death via the extrinsic pathway of apoptosis, Lck-*Cre*-*Apaf1*^f/f^ T cells showed substantial susceptibility to cell death just like control Apaf1-sufficient T cells (Fig 1D). These data collectively demonstrated that Apaf1 is required for intrinsic pathway of apoptosis induced by mitochondrial insult in both thymocytes and T cells and also that Apaf1 is not required for controlling the number of T cells in the thymus and in the lymph nodes.

### Exacerbation of DTH responses in Lck-*Cre*-*Apaf1*^f/f^ mice

**Fig 2:**
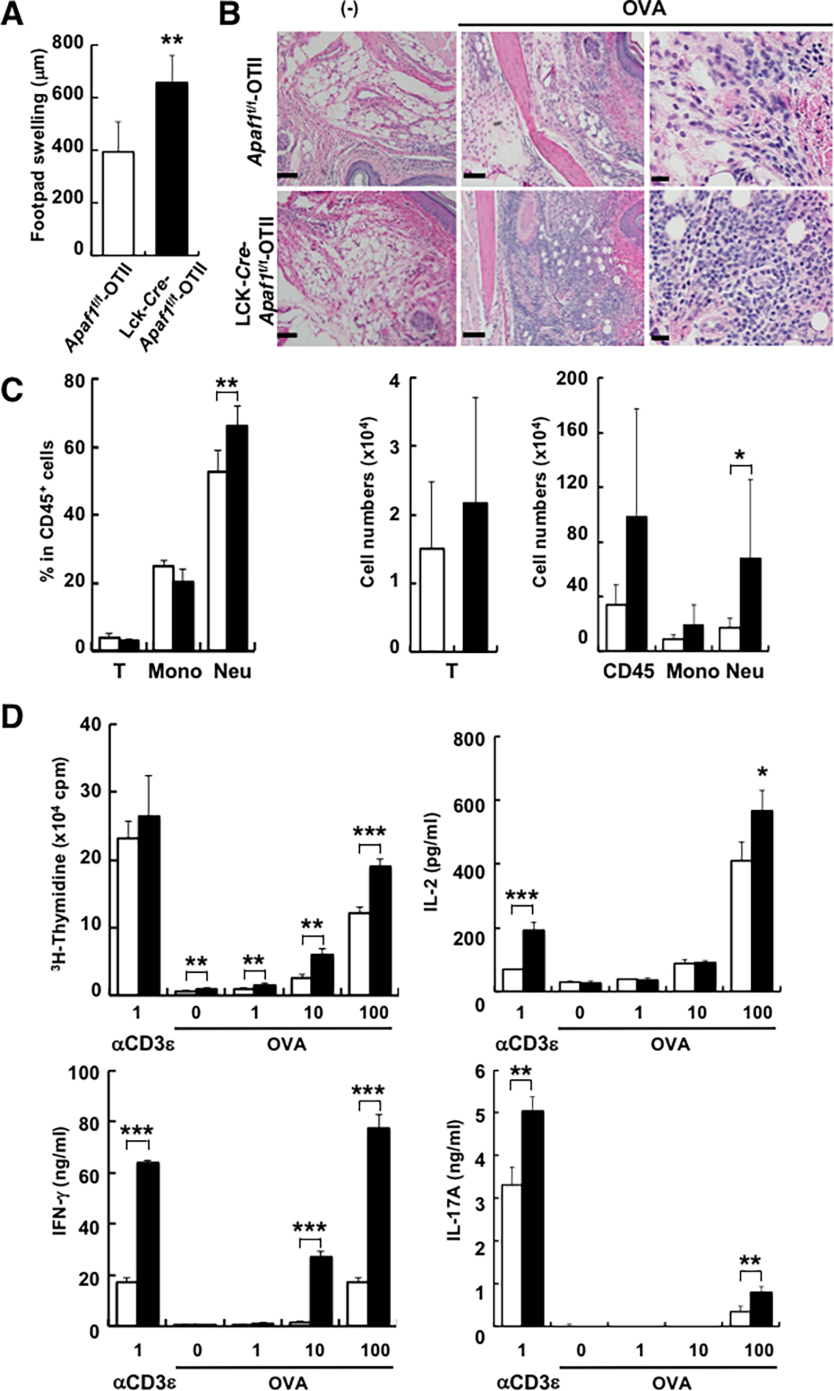
Augmented DTH responses in Lck-*Cre*-*Apaf1*^f/f^ mice. (A) Apaf1f/f-OTII or Lck-*Cre*-*Apaf1*^f/f^ -OTII mice were immunized with OVA and challenged at the footpad 7 days later. Footpad swelling at 48 hours is shown. (B) H&E staining of the footpad lesions. Original magnification; x10 (left and middle, scale bar = 100 μm) and x40 (right, scale bar = 20 μm). (-); challenge without OVA. (C) Percentage and cell number of lesion infiltrating cells. Percentages (left) and cell numbers (middle and right) for T cells (T; CD3^+^), inflammatory monocytes (Mono; CD11b^+^Ly6G^low^), neutrophils (Neu; CD11b^+^Ly6G^high^) and CD45^+^ cells are shown. Open columns; *Apaf1*^f/f^-OTII mice and closed columns; Lck-*Cre*-*Apaf1*^f/f^-OTII mice. (D) LN cells from the two groups of mice were stimulated with OVA or anti-CD3ε antibody and cell proliferation or cytokine production was measured. Shown are mean+SD of triplicated samples. Experiments were repeated three times (n = 6–8 per group) with similar results and representative data are shown. *; p<0.05, **; p<0.01, and ***; p<0.002 compared between *Apaf1*^f/f^ and Lck-*Cre*-*Apaf1*^f/f^ cells, *t*-tests.

Given the role of Apaf1 in the regulation of intrinsic pathway of apoptosis in T cells, we then asked the pathophysiological roles of Apaf1 *in vivo*. To do so, we took advantage of OTII mice, in which T cells express OVA-specific T cell receptors. When control *Apaf1*^f/f^-OTII mice or Lck-*Cre*-*Apaf1*^f/f^-OTII mice were subject to OVA-induced DTH responses, significantly higher levels of footpad swelling were detected in Lck-*Cre*-*Apaf1*^f/f^-OTII mice than in control mice (Fig 2A). Histological examination also revealed higher numbers of infiltrating cells in the footpad lesion in Lck-*Cre*-*Apaf1*^f/f^-OTII mice than in control mice (Fig 2B). Flow cytometric analysis showed that the numbers and the percentages of infiltrating neutrophils were higher in Lck-*Cre*-*Apaf1*^f/f^-OTII mice than control mice while those for lymphocytes and monocytes were comparable (Fig 2C). These observations clearly showed that Apaf1 in T cells was involved in the pathogenesis of DTH responses.

To address the cause of the exacerbated DTH response in Lck-*Cre*-*Apaf1*^f/f^-OTII mice, we performed OVA-specific recall response by T cells *ex vivo*. As shown in Fig 2D, an enhanced proliferative response was observed in Lck-*Cre*-*Apaf1*^f/f^-OTII T cells from the draining LNs, accompanied with significantly higher levels of IL-2 production over control Apaf1-suffucient T cells. DTH response is largely dependent on IFN-γ-producing Th1 cells and, to a lesser degree, on IL-17-producing Th17 cells. As expected, production of both IFN-γ and IL-17A was significantly augmented by Lck-*Cre*-*Apaf1*^f/f^-OTII LN cells as compared with control *Apaf1*^f/f^-OTII LN cells. The levels of IFN-γ and IL-17 indicated that Th1 response was obviously dominant in this experimental system as compared with Th17 response. Higher numbers of infiltrating neutrophils at the footpad lesion in Lck-*Cre*-*Apaf1*^f/f^-OTII mice than control mice, however, implicated involvement of IL-17 in this system. These observations collectively demonstrated that Apaf1 in CD4^+^ T cells critically participated in the attenuation of DTH response.

### Enhanced activation of Apaf1-deficient T cells

**Fig 3:**
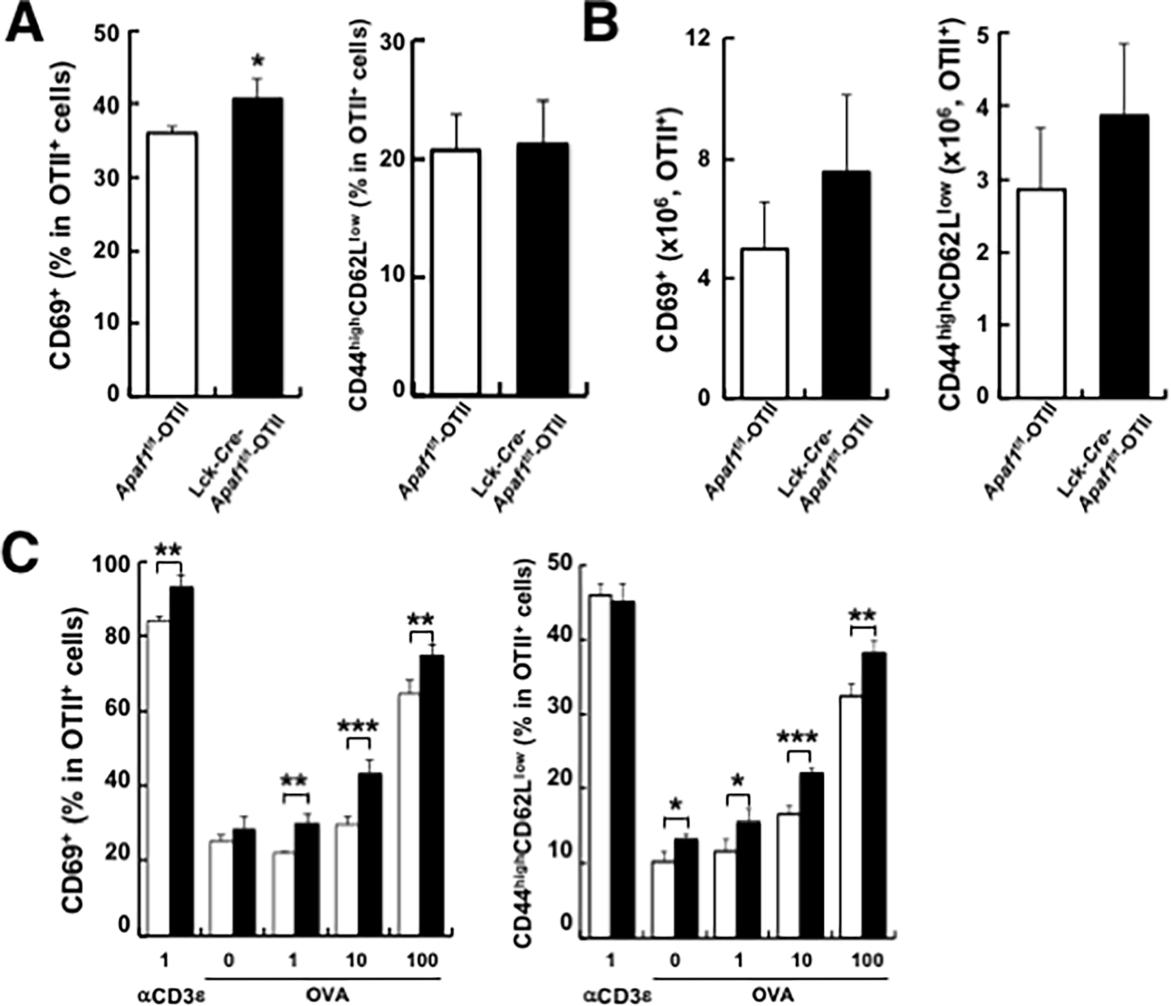
Enhanced *ex vivo* recall responses of Apaf1-deficient T cells. (A and B) LN cells from OVA-immunized *Apaf1*^f/f^-OTII or Lck-*Cre*-*Apaf1*^f/f^-OTII mice were assessed for expression of CD69, CD44 and CD62L. Percentages (A) and absolute numbers (B) of CD69^+^ cells and CD44^high^CD62L^low^ cells in TCR Tg-positive population (OTII population) are shown. (C) LN cells from immunized mice were re-stimulated with OVA or anti-CD3ε antibody at the indicated concentration for 48 hours. Percentages of CD69^+^ cells and CD44^high^CD62L^low^ cells are shown. Open columns; *Apaf1*^f/f^-OTII mice and closed columns; Lck-*Cre*-*Apaf1*^f/f^-OTII mice. Shown are mean+SD of triplicated samples. Experiments were repeated three times with similar results. *; p<0.05, **; p<0.01, and ***; p<0.002 compared between *Apaf1*^f/f^ and Lck-*Cre*-*Apaf1*^f/f^ cells, *t*-tests.

We next examined the expression of early activation marker CD69, and CD44/CD62L as activation/effector markers on T cells from OVA-immunized *Apaf1*^f/f^-OTII and Lck-*Cre*-*Apaf1*^f/f^-OTII mice. The percentages of CD69^+^ OTII cells and those of CD44^high^CD62L^low^ OTII cells as cells with activated (or memory) phenotype in Lck-*Cre*-*Apaf1*f/f-OTII mice were comparable to those in *Apaf1*^f/f^-OTII mice (Fig 3A). Similarly, the total numbers of CD69^+^ cells and CD44^high^CD62L^low^ cells were comparable between the two groups (Fig 3B). We then evaluated these cell surface markers following the re-stimulation of T cells *in vitro*. As expected, the expression of activated marker CD69 rapidly up-regulated upon re-stimulation on both Lck-*Cre*-*Apaf1*^f/f^-OTII and control cells in an antigen dose-dependent manner (Fig 3C, left). Expression of CD69 was higher on Lck-*Cre*-*Apaf1*^f/f^-OTII cells than on control cells (Fig 3C, left and Panel A in S3 Fig). The percentages of CD44^high^CD62L^low^ cells also increased after *in vitro* re-stimulation and were higher in Lck-*Cre*-*Apaf1*^f/f^-OTII cells than in control Apaf1-sufficient cells (Fig 3C, right and Panel B in S3 Fig).

**Fig 4:**
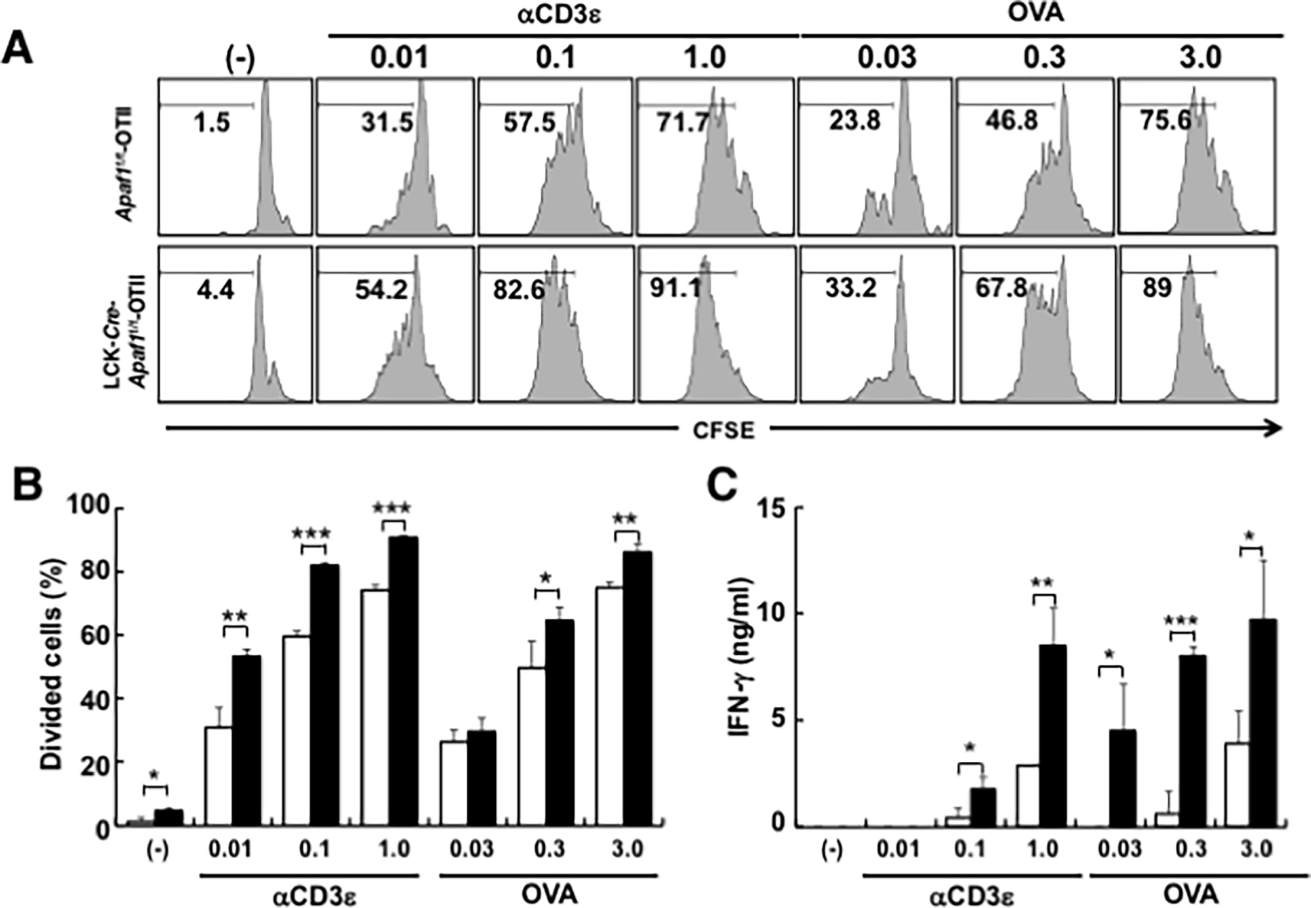
Enhanced proliferation and cytokine production by Apaf1-deficient naïve T cells. (A and B) LN cells from unimmunized *Apaf1*^f/f^-OTII or Lck-*Cre*-*Apaf1*^f/f^ -OTII mice were labeled with CFSE and stimulated with anti-CD3ε antibody or OVA peptide at the indicated concentrations. CFSE intensity was analyzed and cells with lower intensity (in cell division) were measured. Representative figures with percentages of cells with lower CFSE intensity are shown in A and results with triplicated samples are shown in B. (C) IFN-γ production by LN cells from unimmunized mice stimulated with anti-CD3ε antibody or OVA peptide. Open columns; *Apaf1*^f/f^-OTII mice and closed columns; Lck-*Cre*-*Apaf1*^f/f^-OTII mice. Experiments were repeated three times with similar results. Shown are mean+SD. Experiments were repeated three times with similar results. *; p<0.05, **; p<0.01, and ***; p<0.002 compared between *Apaf1*f/f and Lck-*Cre*-*Apaf1*^f/f^ cells, *t*-tests.

We further addressed the impact of Apaf1-deficiency in primarily activated T cells. When CD4^+^ T cells from unimmunized mice were stimulated *in vitro* with either OVA peptide or anti-CD3ε antibody, Lck-*Cre*-*Apaf1*^f/f^-OTII cells showed higher or more efficient cell division than control cells, as demonstrated by higher percentages of cells with lower CFSE intensity (Fig 4A). Percentages of divided cells with lower CFSE intensity were significantly higher in Apaf1-deficient Lck-*Cre*-*Apaf1*^f/f^-OTII cells than in Apaf1-sufficient control cells (Fig 4B). Production of IFN-γ in the supernatants was also significantly higher by Lck-*Cre*-*Apaf1*^f/f^-OTII cells than by control *Apaf1*^f/f^-OTII cells (Fig 4C). These data shown in Figs 3 and 4 clearly demonstrated that Apaf1 in T cells plays a regulatory role during an initial encounter and activation of naive T cells as well as during recall activation of primed T cells with antigen.

### Caspase-independent enhancement of activation in Apaf1-deficient T cells

**Fig 5:**
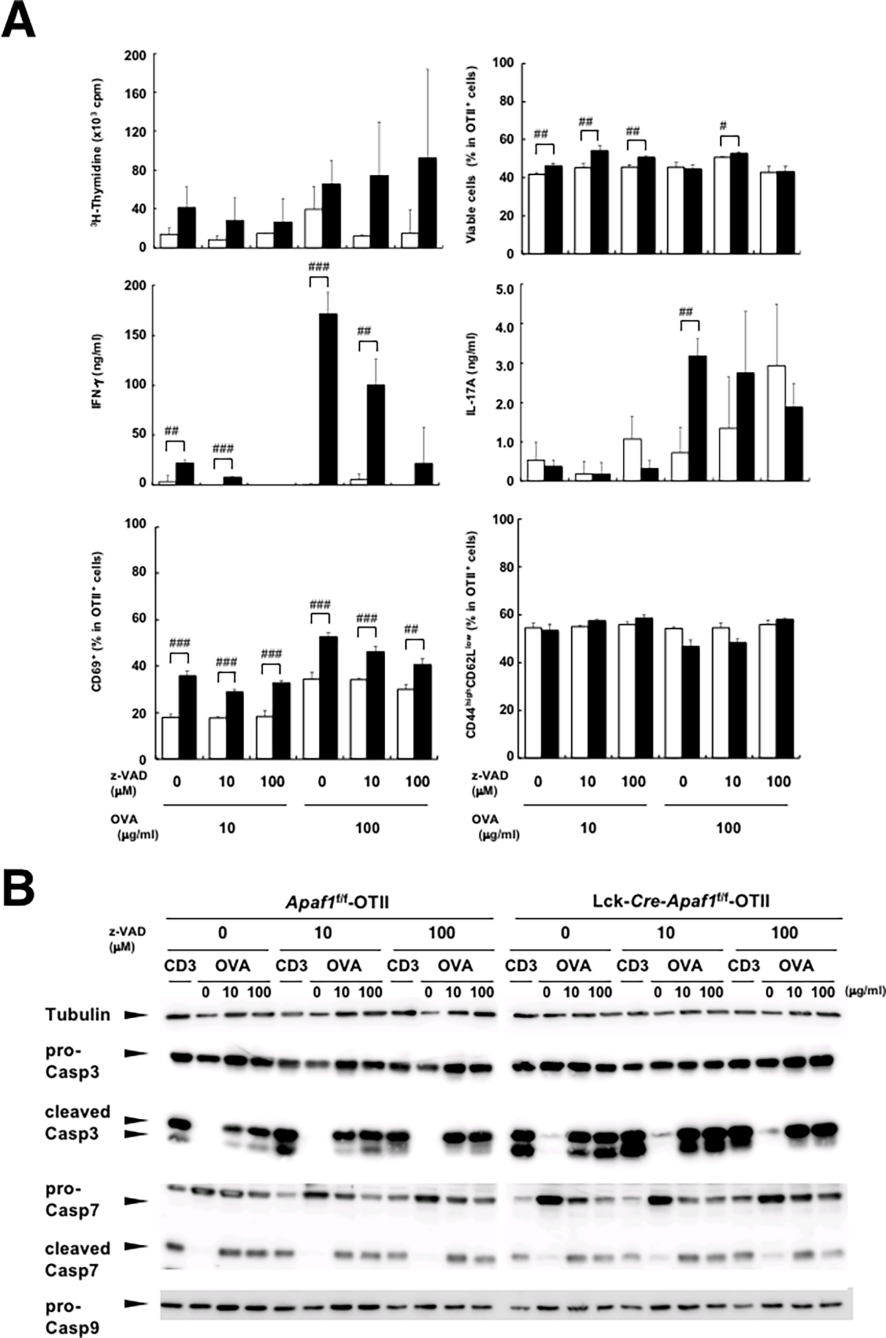
Caspase-independent role of Apaf1 in T cell activation. LN cells from immunized *Apaf1*^f/f^-OTII or Lck-*Cre*-*Apaf1*^f/f^ -OTII mice were stimulated with indicated doses of OVA or anti-CD3ε antibody in the presence or absence of z-VAD-*fmk* (z-VAD) for 48 hours. (A) Cell proliferation, cell viability (Annexin V-negative and PI-negative), production of IFN-γ and IL-17, or expression of CD69, CD44, and CD62L were analyzed. Open columns; *Apaf1*^f/f^-OTII mice and closed columns; Lck-*Cre*-*Apaf1*^f/f^-OTII mice. Data show means + SD of triplicated samples. Experiments were repeated three times with similar results. #; p’<0.05, ##; p’<0.01, and ###; p’<0.002, *t*-tests with Bonferroni correction compared among indicated samples. (B) Cell lysates were prepared from cells in A, electrophoresed, and blotted. Pro- or cleaved form of caspase 3 and 7, and pro-caspase 9 were detected with respective antibodies. Tubulin was detected as an internal control. Experiments were repeated two times with similar results.

The negative regulation of T cell activation by Apaf1 may or may not depend on apoptosis. T cells activated in an inappropriate condition may be deleted by Apaf1-dependent apoptosis. Apaf1 may, however, regulate T cell activation and proliferation independently of caspase activation/apoptosis. Unexpectedly, pan-caspase inhibitor, z-VAD-*fmk*, at 10 or 100 μM did not rescue the lower proliferation (thymidine incorporation) or lower viability of control Apaf1-sufficient cells over Apaf1-deficient cells from immunized mice after antigenic stimulation (Fig 5A, upper panels). Similarly, neither lower production of IFN-γ nor IL-17 production by Apaf1-sufficient cells were restored to the levels by Apaf1-deficient cells with z-VAD-*fmk* (Fig 5A, middle panels). Additionally, percentages of CD69^+^ and CD44^high^CD62L^low^ cells in control Apaf1-sufficient OTII T cell population were still lower over Apaf1-deficient OTII T cells in the presence of z-VAD-*fmk* (Fig 5A, lower panels). Dexamethasone-induced apoptosis and caspase 3 activation in thymocytes was completely suppressed by z-VAD-*fmk* at the same concentration (100 μM, S4 Fig).

To address whether or not caspase activation occurs during T cell activation, either in Apaf1-dependent or -independent way, western blot analysis was performed to detect caspase activation during antigen-induced T cell activation (Fig 5B). Stimulation of LN T cells with anti-CD3ε antibody or OVA induced caspase 3 cleavage in *Apaf1*^f/f^-OTII cells. Perhaps counterintuitively, but in line with the data show above, caspase 3 cleavage was similarly detected in Lck-*Cre*-*Apaf1*^f/f^-OTII cells. The caspase inhibitor, z-VAD-*fmk*, did not inhibit caspase 3 cleavage in either Apaf1-sufficient or Apaf1-deficient cells, although the appearance of 17 kDa fragment was only partially inhibited by z-VAD-*fmk* at 100 μM (Fig 5B, cleaved Casp3). Cleaved form of caspase 7 was also detected in both Apaf1-sufficient T cells and Apaf1-deficient T cells almost similarly (cleaved Casp7) and z-VAD-*fmk* at 100 μM showed no inhibitory effects. Caspase 9 activation (cleavage), which should be shown by reduction of pro-caspase 9, was barely detectable in both Apaf1-sufficient or -deficient OTII cells; note that cleaved form of caspase 9 was not detectable with antibody used. The western blot analysis clearly demonstrated that antigen-specific activation of T cells induced caspase 3 and 7 cleavage, which was not mediated by Apaf1 (and presumably not by caspase 9) and also was not inhibited by z-VAD-*fmk*. As the caspase cleavage occurred similarly in both Apaf1-sufficient and Apaf1-deficient T cells, caspase activation did not account for the difference in proliferation and cytokine production between Apaf1-sufficient and Apaf1-deficient T cells. These data indicated that the negative impact of Apaf1 on antigen-stimulated T cells was largely independent of caspase activities, although the possible involvement of caspase/apoptosis was not formally excluded.

Taken together, our data clearly demonstrated that Apaf1 is a negative regulator of T cell responses by attenuating proliferation, activation, and cytokine production. This negative regulatory role of Apaf1 plays a role during antigen-specific T cell response but not in the maintenance of T cell homeostasis and the role is caspase-independent.

## Discussion

Previous reports including ours demonstrated the role of Apaf1 in the functional shaping of some organs and adjusting the number of cells during development[9, 10, 14–16]. While apoptosis is required for removal of unwanted or possibly hazardous immune cells, the negative selection of self-reactive clones in the thymus was not impaired in Apaf1-deficient mice [6]. In addition, while almost all Apaf1-deficient mice were perinatally lethal due to the brain deformity, a few survivors observed showed no signs of lymph adenopathy (H. Y., unpublished observation), implying no role of Apaf1 in the T cell homeostasis in the periphery. In the current study, we addressed the role of Apaf1 in regulation of antigen-specific T cell responses. Perinatal lethality of Apaf1-deficient mice prevented us from examining the role of Apaf1 in the T cell activation. To circumvent this problem, we generated conditional Apaf1-deficient mice, in which Lck-driven expression of the Cre recombinase disrupted *Apaf1* gene in a T cell-specific manner. Data shown in Fig 1 confirmed the requirement of Apaf1 for the intrinsic pathway of apoptosis, induced by mitochondrial damages including growth factor deprivation, but not for the extrinsic pathway of apoptosis, induced by, for instance, anti-CD3ε re-stimulation or Fas ligation (data not shown), in the thymocytes and peripheral T cells.

To examine the role of Apaf1 in T cells during antigen-induced immune responses, we exploited OVA-specific delayed-type hypersensitivity assay using OTII mice expressing OVA-specific T cell receptors on T cells. Lck-*Cre*-*Apaf1*^f/f^-OTII mice showed remarkable exacerbation of DTH responses including footpad swelling, cell infiltration, and inflammatory cytokine production (Fig 2), clearly demonstrating the role of Apaf1 as a negative regulator of T cell responses. To our knowledge, this is the first to show the role of Apaf1 as a negative regulator of immune responses. Apoptosis occurs in mature T lymphocytes in an antigen-driven way or growth factor-deprived way and is required for homeostasis, tolerance, and proper regulation of immune responses [17]. There are a couple of different (but not mutually exclusive) possibilities where Apaf1 plays its negative role during T cell responses. One is that Apaf1-deficiency leads to prolonged survival of T cells after activation, due to the impairment of Apaf1-dependent apoptosis, resulting in the augmentation of DTH responses. Although this possibility appeared plausible, we were unable to detect any difference in the percentages of apoptotic cell death in the infiltrating cells into the footpad lesion between Lck-*Cre*-*Apaf1*^f/f^-OTII and *Apaf1*^f/f^-OTII mice (data not shown). In addition, time course of the footpad swelling between the two groups of mice were almost comparable (data not shown). It was thus unlikely that Apaf1 attenuated T cells responses by controlling apoptosis, although the possibility was not formally excluded.

Our *ex vivo* and *in vitro* analyses of antigen-specific cell activation supported another possibility that Apaf1 regulates T cell activation, independently of cell survival. Apaf1-deficient OTII T cells upon stimulation proliferated more efficiently and showed higher percentages of cells with activation phenotypes (Figs 3 and 4 and S3 Fig). Although these differences in activation status could be explained by the impairment of Apaf1-dependent apoptosis in inappropriately activated T cells, addition of a pan-caspase inhibitor, z-VAD-*fmk*, did not affect the difference in activation and cell division of T cells *in vitro* (Fig 5A). It was thus indicated that the effect of Apaf1 in regulation of cell activation and cell division was caspase-independent. As reported previously [18], caspase 3 was cleaved during T cell activation, which was not inhibited by z-VAD-*fmk*. We also detected caspase 3 and 7 cleavage during T cell activation, on which z-VAD-*fmk* showed little, if any, inhibitory effects (Fig 5B). The cleavage of caspases 3 and 7 occurred similarly in both Apaf1-sufficient and Apaf1-deficient T cells. As caspase 9 activation was barely detectable in these cells, the cleavage of caspases 3 and 7 appeared independent of apoptosome activation. Although the precise mechanism for this caspase activation is not known yet, it is clear that the caspase activation was not the reason for the difference in T cell activation between Apaf1-sufficient and -deficient T cells. The roles of Apaf1 reside mainly but are not limited in the initiation of apoptosis. Zermati et al. reported the non-apoptotic role of Apaf1 in that Apaf1-deficiency resulted in the impairment of DNA damage-induced cell cycle arrest[19]. This non-apoptotic function of Apaf1 requires nuclear translocation of Apaf1 and depends of checkpoint kinase-1 (Chk1) activity. Similarly, chemical inhibitor of Apaf1 suppressed Apaf1-dependent DNA-damage checkpoint[20]. These reports supported anti-proliferation and/or anti-survival function of Apaf1. Oppositely, Apaf1 plays a pro-proliferation/pro-survival role by regulating centrosome stability. Ferraro et al. reported that Apaf1 was required for efficient centrosome and mitotic spindle formation[21]. The non-apoptotic roles of Apaf1 are thus complicated and perhaps context-dependent[22]. Although the precise mechanisms for these non-apoptotic functions of Apaf1 are poorly understood, Apaf1 exerts these functions through interaction with various molecules[21, 23–29]. Presumably, Apaf1 plays its anti- or pro-activation/survival function though interaction with the interacting molecule(s) depending upon cell activation status. In our hands also, Apaf1-deficiency in some situations resulted in enhanced cell death (e.g. Fig 1C, Fas stimulation). This may reflect the context-dependent (non-apoptotic) roles of Apaf1.

We were unable to detect any difference in cell cycle progression or cell activation status in the lesion-infiltrating T cells between Apaf1-deficient and Apaf1-sufficient cells in DTH assay (data not shown). It is possible that the difference in individual cells was subtle and/or only a small fraction of antigen-specifically activated cells showed difference. Alternatively, it is possible that Apaf1 exerts its negative regulatory effect at the priming phase where naive T cells are primarily stimulated in the draining lymph nodes, before the effector phase. Higher percentages of cells in activation status in Apaf1-deficient T cells at the primary stimulation phase *in vitro* (Fig 4) may support this idea. In any case, the impact of Apaf1-deficiency on T cell function appeared limited. We have been unable to detect significant differences in pathological phenotypes in other disease models, including experimental allergic encephalomyelitis (EAE) and LPS-induced topical inflammation (data not shown). In DTH model in the current study, the differences were small albeit their significance and reproducibility. In our hands, we reported that the DTH response was exacerbated in the absence of EBI-3 of IL-27 [30]. The phenotypic difference was far worse in the EBI-3-deficient mice than in T cell-specific Apaf1-deficient mice (compared data not shown). The role of Apaf1 and Apaf1-mediated apoptosis in T cell function appears much limited in terms of the intensity and/or duration, as compared with, for instance, cytokines or other molecules directly involved in T cell activation/function. Further clarification of the exact point(s) where Apaf1 exerts it regulatory effect during T cell activation is a future challenge.

Collectively, our data clearly demonstrated Apaf1 as a negative regulator of T cell responses. Further analysis of the underlying mechanisms may reveal Apaf1 as a potential target for immunosuppressive drug discovery.

## Supporting information

S1 Fig Generation of *Apaf1* mutant mice. A part of wild-type *Apaf1* allele, structure of the targeting vector, and the resultant mutated allele with (*Apaf1*^f-Neo^) or without (*Apaf1*^flox^) neomycin resistant gene are shown. DTA; diphtheria toxin fragment A gene, Neo; neomycin resistance gene, Flpe; FLPe recombinase (treatment or expression). Two probes used for confirmation of homologous recombination (ex probe 1 and 2) and a probe used for the genomic Southern blot (Fig 1A) are shown.
(TIFF)

S2 Fig Resistance to growth factor-deprivation and susceptibility to activation-induced cell death of Apaf1-deficient T cells. Cells were prepared as in Fig 1D and flow cytometric analysis data are shown; CM; conditioned medium (from primary stimulation culture), Media; fresh medium, αCD3ε; anti-CD3ε stimulation. Percentages of Annexin V-negative and PI-negative viable cells (left lower quadrant) and Annexin V-positive and PI-positive dead cells (right upper quadrant) are shown.
(TIFF)

S3 Fig Enhanced *ex vivo* recall responses of Apaf1-deficient T cells. LN cells from OVA-immunized *Apaf1*^f/f^-OTII or Lck-*Cre*-*Apaf1*^f/f^ -OTII mice were assessed as in Fig 3C. Representative figures are shown. Percentages of CD69^+^ cells (A) and of CD44^high^CD62L^low^ cells (B, right lower quadrant) are shown.
(TIFF)

S4 Fig Successful inhibition of dexamethasone-induced apoptosis by z-VAD-*fmk*. (A) Thymocytes were prepared from wild-type C57BL/6 mice and treated with indicated doses of dexamethasone (Dex, nM) in the absence (-) or presence of DMSO (D) or 100 μM of z-VAD-*fmk* (100). Apoptotic cells were evaluated by Annexin V and PI staining. (B) Cell lysates were prepared from thymocytes in A (Dexamethasone; 100 nM), electrophoresed, and blotted. Caspase 3 (pro- and cleaved form) were detected with anti-Casp3 antibody. Tubulin was detected as a control.
(TIFF)
