# Supplementary figures and images for: Apaf1 plays a negative regulatory role in T cell responses by suppressing activation of antigen-stimulated T cells
Source: PLoS One. 2018 Mar 29;13(3):e0195119. doi: 10.1371/journal.pone.0195119 (PMC5875858; doi:10.1371/journal.pone.0195119)

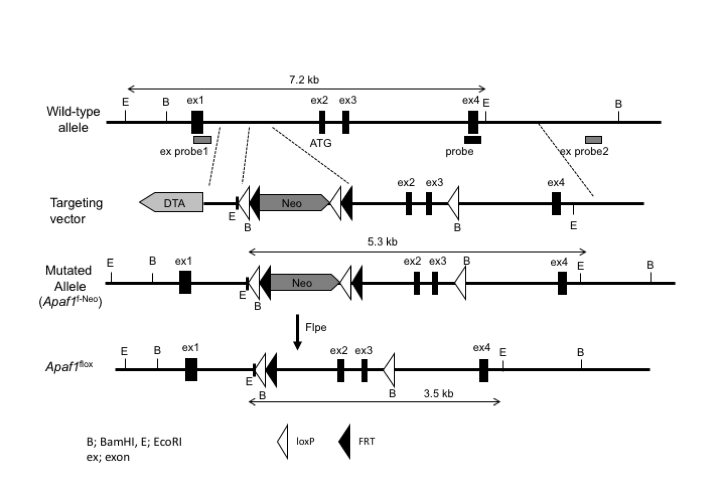

Supplement: S1 Fig — A part of wild-type Apaf1 allele, structure of the targeting vector, and the resultant mutated allele with (Apaf1f-Neo) or without (Apaf1flox) neomycin resistant gene are shown. DTA; diphtheria toxin fragment A gene, Neo; neomycin resistance gene, Flpe; FLPe recombinase (treatment or expression). Two probes used for confirmation of homologous recombination (ex probe 1 and 2) and a probe used for the genomic Southern blot (Fig 1A) are shown. (TIFF) [file pone.0195119.s001.tiff]

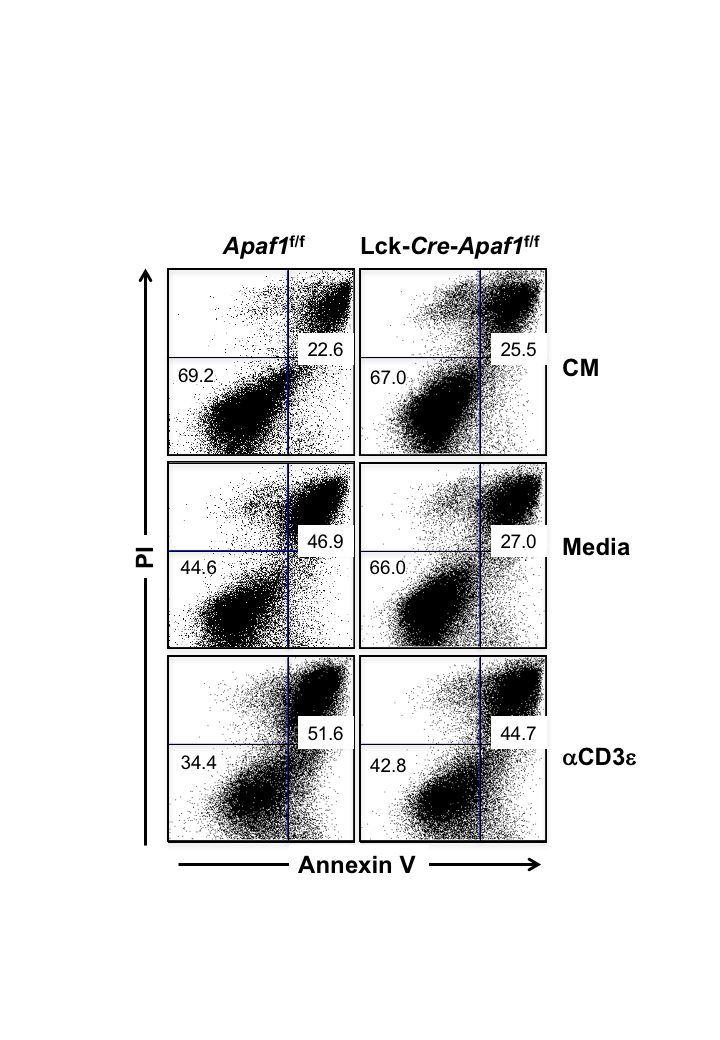

Supplement: S2 Fig — Cells were prepared as in Fig 1D and flow cytometric analysis data are shown; CM; conditioned medium (from primary stimulation culture), Media; fresh medium, αCD3ε; anti-CD3ε stimulation. Percentages of Annexin V-negative and PI-negative viable cells (left lower quadrant) and Annexin V-positive and PI-positive dead cells (right upper quadrant) are shown. (TIFF) [file pone.0195119.s002.tiff]

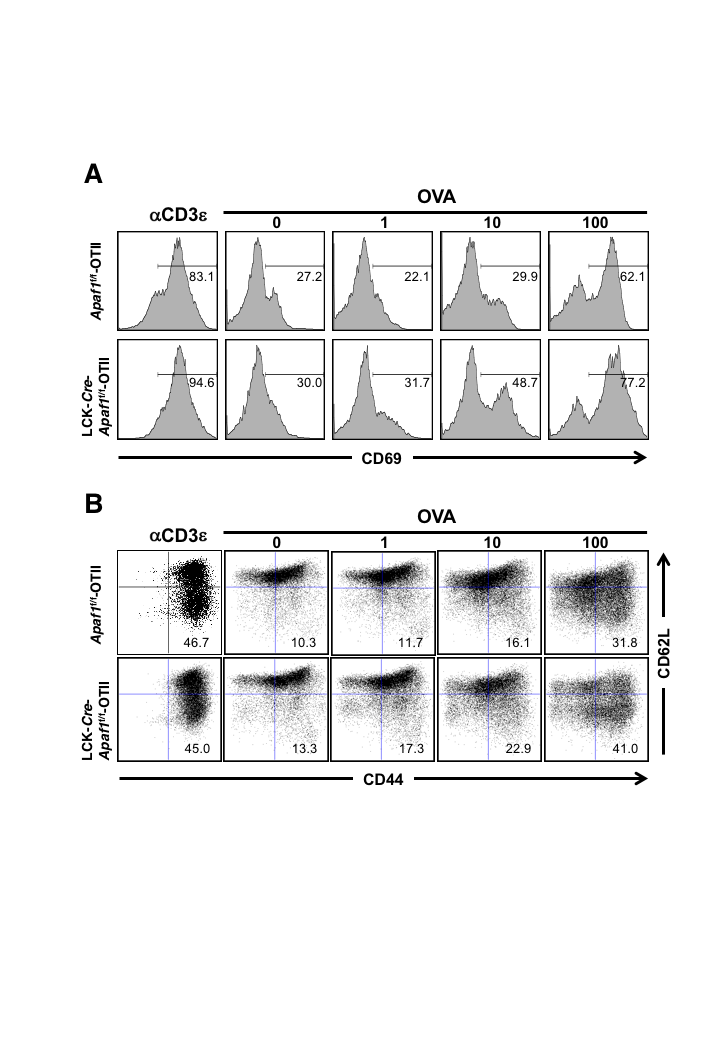

Supplement: S3 Fig — LN cells from OVA-immunized Apaf1f/f-OTII or Lck-Cre-Apaf1f/f -OTII mice were assessed as in Fig 3C. Representative figures are shown. Percentages of CD69+ cells (A) and of CD44highCD62Llow cells (B, right lower quadrant) are shown. (TIFF) [file pone.0195119.s003.tiff]

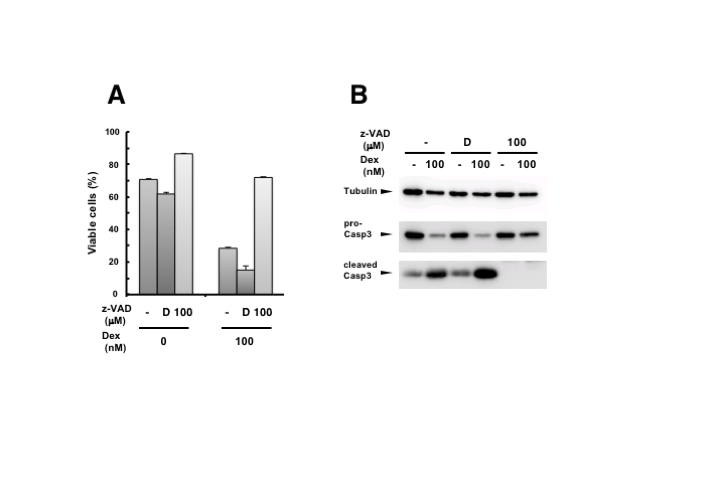

Supplement: S4 Fig — (A) Thymocytes were prepared from wild-type C57BL/6 mice and treated with indicated doses of dexamethasone (Dex, nM) in the absence (-) or presence of DMSO (D) or 100 μM of z-VAD-fmk (100). Apoptotic cells were evaluated by Annexin V and PI staining. (B) Cell lysates were prepared from thymocytes in A (Dexamethasone; 100 nM), electrophoresed, and blotted. Caspase 3 (pro- and cleaved form) were detected with anti-Casp3 antibody. Tubulin was detected as a control. (TIFF) [file pone.0195119.s004.tiff]
